# Phosphoserine phosphatase as a prognostic biomarker in patients with gastric cancer and its potential association with immune cells

**DOI:** 10.1186/s12876-021-02073-0

**Published:** 2022-01-03

**Authors:** Ma-Yan Huang, Xiao-Yun Liu, Qiong Shao, Xu Zhang, Lei Miao, Xiao-Yan Wu, Yu-Xia Xu, Fang Wang, Hai-Yun Wang, Liang Zeng, Ling Deng

**Affiliations:** 1 Department of Pathology, Sun Yat-Sen University Cancer Center, State Key Laboratory of Oncology in South China, Collaborative Innovation Center for Cancer Medicine, Guangzhou, 510060 Guangdong People’s Republic of China; 2 Department of Molecular Diagnostics, Sun Yat-Sen University Cancer Center, State Key Laboratory of Oncology in South China, Collaborative Innovation Center for Cancer Medicine, Dongfeng East Road 651, Guangzhou, 510060 Guangdong People’s Republic of China; 3 Department of Pediatric Surgery, Guangzhou Institute of Pediatrics, Guangdong Provincial Key Laboratory of Research in Structural Birth Defect Disease, Guangzhou Women and Children’s Medical Center, Guangzhou Medical University, Guangzhou, 510623 People’s Republic of China; 4 Department of Pathology, Guangzhou Women and Children’s Medical Center, Guangzhou Medical University, No. 9 Jinsui Road, Guangzhou, 510623 People’s Republic of China; 5 Guangzhou Institute of Pediatrics, Guangdong Provincial Key Laboratory of Research in Structural Birth Defect Disease, Guangzhou Women and Children’s Medical Center, Guangzhou Medical University, Guangzhou, 510623 People’s Republic of China

**Keywords:** PSPH, Metabolic genes, Poor prognosis, Immune score, Gastric cancer

## Abstract

**Background:**

Because of dismal prognosis in gastric cancer, identifying relevant prognostic factors is necessary. Phosphoserine phosphatase (PSPH) exhibits different expression patterns in many cancers and has been reported to affect the prognosis of patients with cancer. In this study, we examined the prognostic role of metabolic gene PSPH in gastric cancer based on the TCGA dataset and our hospital–based cohort cases.

**Methods:**

We collected and analysed RNA-seq data of Pan-cancer and gastric cancer in the TCGA dataset and PSPH expression data obtained from immunohistochemical analysis of 243 patients with gastric cancer from Sun Yat-sen University cancer center. Further, Kaplan–Meier survival analysis and Cox analysis were used to assess the effect of PSPH on prognosis. The ESTIMATE and Cibersort algorithms were used to elucidate the relationship between PSPH and the abundance of immune cells using the TCGA dataset.

**Results:**

We observed that PSPH expression displayed considerably high in gastric cancer and it was significantly associated with inferior prognosis (*P* = 0.043). Surprisingly, there was a significant relationship between lower immune scores and high expression of PSPH (*P* < 0.05). Furthermore, patients with a low amount of immune cells exhibited poor prognosis (*P* = 0.046). The expression of PSPH significantly increased in activated memory CD4 T cells, resting NK cells and M0 macrophages (*P* = 0.037, < 0.001, and 0.005, respectively).

**Conclusions:**

This study highlighted that PSPH influences the prognosis of patients with gastric cancer, and this is associated with the infiltration of tumour immune cells, indicating that PSPH may be a new immune-related target for treating gastric cancer.

**Supplementary Information:**

The online version contains supplementary material available at 10.1186/s12876-021-02073-0.

## Background

Gastric cancer is one of the most well-known cancers of the gastrointestinal tract. In China, it is ranked third as far as incidence rate is concerned and is a major cause of cancer mortality [1]. Systematic treatment regimens are being considerably improved in clinical practice; however, the prognosis of gastric cancer still remains unfavourable because of poor recognition and suboptimal risk stratification of the preneoplastic condition, atrophic gastritis, and aggressive cancer behaviour [2, 3]. Lauren’s classification is the most useful and widely applicable classification for pathology, generally dividing the disease into intestinal-, diffuse-, and mixed-subtype [4]. Although a few of molecular biomarkers have been used for predicting prognosis to date [5, 6], few of clinical and prognostic biomarkers are available in gastric cancer. Therefore, searching biomarkers for prognosis prediction in gastric cancer patients could be helpful in deciding more effective clinical regimen.

The deregulation of metabolic pathways fuels cell metastasis during tumorigenesis, possibly contributing to the poor prognosis in gastric cancer. Phosphoserine phosphatase (PSPH) is an enzyme, which is involved in the process of L-serine biosynthesis. Recent studies indicated that PSPH mainly plays role in multiple aspects of cell behaviours such as proliferation and differentiation by producing precursors for the biosynthesis of diverse compounds including neurotransmitters, glycolipids and thymidine [7, 8]. Additionally, several studies reported that augmented PSPH level is correlated with the prognosis in multiple cancers including cutaneous squamous cell carcinoma [9], breast cancer [10], non-small cell lung cancer [11], colorectal cancer [12] and hepatocellular carcinoma [13]. This indicates that PSPH may serve as a biomarker for prognosis in cancers. However, very little is known about the prognostic role of PSPH in patients with gastric cancer.

In this study, we analysed PSPH expression across multiple cancers using Pan-Cancer data and focused on the relationship between PSPH expression and gastric cancer. We further demonstrated the differential expression of PSPH in the intestinal- and diffuse-type gastric cancer through immunohistochemistry. Moreover, we observed that patients with high expression of PSPH had inferior survival because of low infiltration of immune cells. In summary, PSPH may help to predict prognosis in patients with gastric cancer and is associated with immune cells.

## Methods

### Clinical gastric cancer specimens

The formalin-fixed paraffin-embedded (FFPE) human gastric cancer tissues for routine pathological diagnosis and immunohistochemical analysis were collected from the Department of Pathology, Sun Yat-sen University Cancer Center (SYSUCC) from January 2014 to December 2016. The disease stage of all patients was classified according to the 8th edition of AJCC staging system [14]. The clinicopathological characteristics of 234 patients with gastric cancer are listed in Table 1.

**Table 1:** Clinicopathologic characteristics of 234 gastric cancers

Variables	No. of patients	%
Age (years)		
Median (range)	59 (22–83)	
Gender		
Female	82	35.0
Male	152	65.0
Lauren classification		
Intestinal-type	64	27.4
Diffuse-type	111	47.4
Mixed	59	25.2
Invasion depth		
T1	25	10.8
T2	23	9.9
T3	93	40.1
T4	91	39.2
Unknown	2	
Lymph node status		
Negative	62	27.2
Positive	166	72.8
Unknown	6	
Distant metastasis		
Negative	208	88.9
Positive	26	11.1
Stage		
I + II	91	39.1
III + IV	142	60.9
Unknown	1	

### Immunohistochemical staining

Immunohistochemical staining was performed to examine the PSPH expression in primary tumour tissues of gastric cancer. The FFPE tissue sections were treated with citrate antigen repair buffer (pH 6.0) and subjected to endogenous peroxidase blocking with 3% hydrogen peroxide. Further, they were incubated with the indicated primary PSPH antibody with 1:100 dilution (ab224110, Abcam, Cambridge, UK) overnight at 4 °C in a humidified chamber. Three sophisticated pathologists (L.Z, X.Z and Q.S) who were blind to the clinical features of specimens independently determined consensus scoring of PSPH expression using a semi-quantitative estimation. Briefly, the proportion of stained tumour cells was assigned as follows: 0, negative; 1, less than 30% of tumour cells were stained, and 2, more than 30% of tumour cells were stained. Staining intensity score was assigned as follows: 0, negative; 1, weak; 2, moderate and 3, strong. The multiple scores were classified as follows: 0–1 was low, and 2–6 was high.

### Data sources and pre-processing

The Gene Expression of pan cancer (PanCanAtlas: http://xena.ucsc.edu/) was downloaded for PSPH expression among 32 cancer types. The cohort of gastric cancer was originated from GDC TCGA Stomach Cancer (STAD) with dataset ID ‘TCGA-STAD.htseq_counts.tsv’, which were downloaded from UCSC Xena (https://gdc-hub.s3.us-east-1.amazonaws.com/download/TCGA-STAD.htseq_counts.tsv.gz; Full metadata). This data of gene expression derived from RNA sequencing were normalised using the Robust Multi-array Average method.

### ESTIMATE evaluation and differential gene enrichment analysis

The immune score of cases was calculated using the ESTIMATE algorithm via the ‘estimate’ package 1.0.13 of R software [15]. A high score indicates a large proportion of the corresponding component in the tumour immune microenvironment. Differential analysis was performed using the DESeq2 package. Cibersort algorithm [16] was applied to analyse the correlations between PSPH expression and 22 immune cell subsets.

### Statistical analysis

The R programming language (https://www.r-project.org/) and Stata version 15.1 (Texas, USA) were applied to visualise the results of data analysis. The correlation between the PSPH expression level and clinicopathological features was assessed using the Fisher’s exact test or the χ^2^ test. Survival curves of patients from STAD and SYSUCC datasets were estimated by Kaplan–Meier analysis with the log-rank test. All statistical tests were two sided and considered significant when the *P* value was less than 0.05.

## Results

### PSPH expression varies as per the sub-type in gastric cancer

**Fig. 1:**
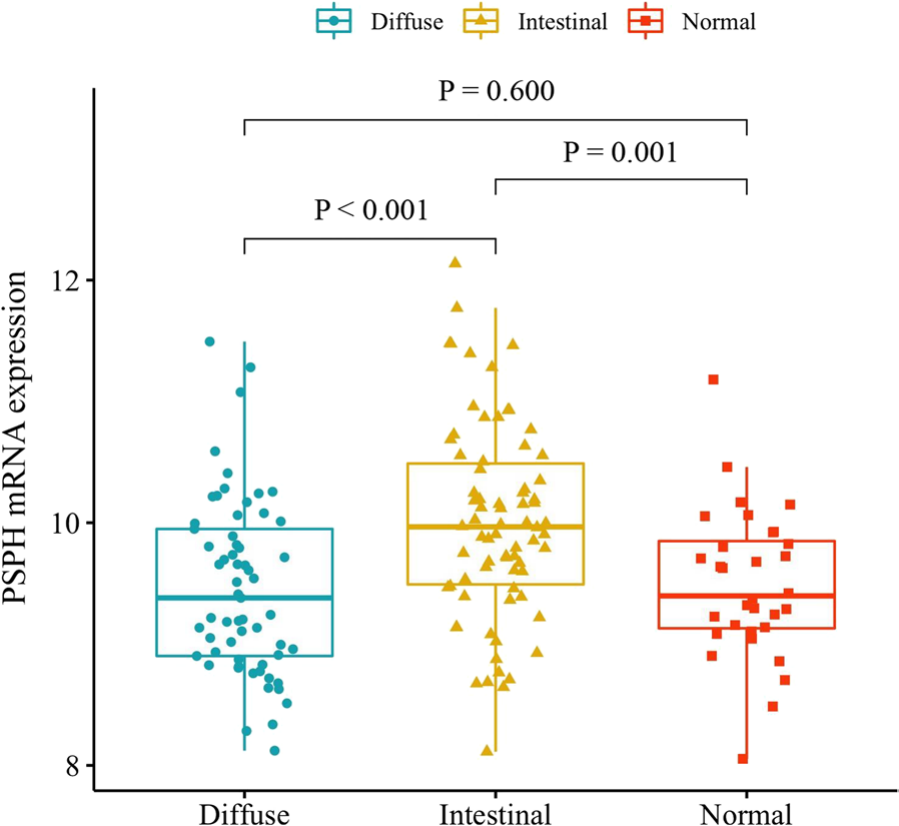
The expression of PSPH in patients with gastric cancer and normal individuals in TCGA-STAD dataset

**Fig. 2:**
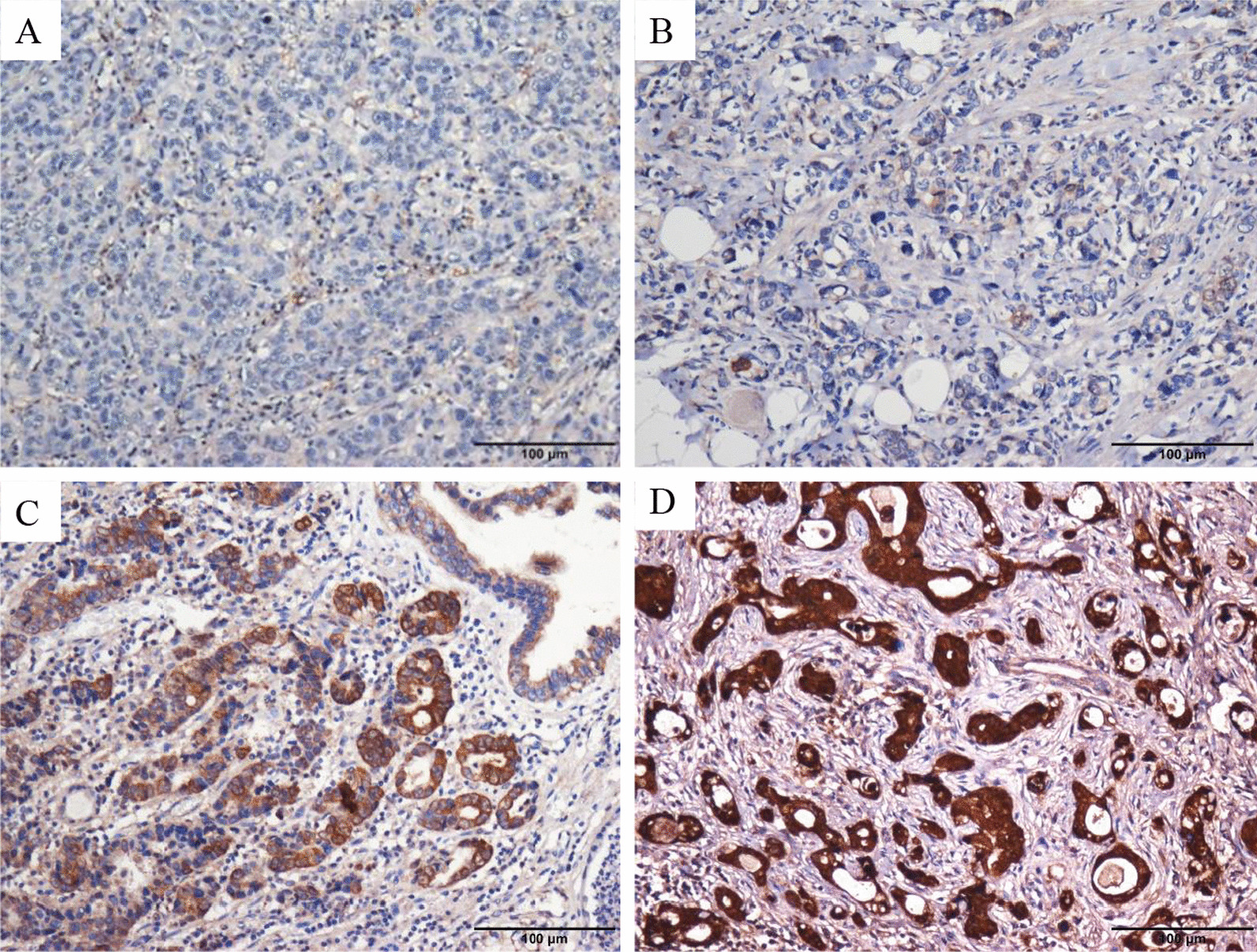
The representative images of PSPH expression detected by immunohistochemistry in patients with gastric cancer: negative (**A**), less (**B**), moderate (**C**) and strong staining (**D**)

In the TCGA-generated RNA-seq data on 9,704 cancer samples of 32 human cancer types (Additional file 2: Table S1), we observed a substantially different expression of PSPH among cancer types (Additional file 1: Figure S1). This differential expression pattern indicated the biological significance of PSPH across multiple cancers. To assess the expression of PSPH in gastric cancer, we further analysed TCGA-STAD cohort data. Interestingly, the mRNA level of PSPH was significantly higher in patients with intestinal-type gastric cancer than in normal individuals and patients with diffuse-type gastric cancer (Fig. 1, *P* = 0.001 and < 0.001, respectively). Further, we carried out immunohistochemical analysis to detect the PSPH expression in gastric cancer (Fig. 2). In the intestinal subtype, high expression of PSPH was seen in 32.5% of patients, whereas low expression was displayed in 64.8% of the diffuse subtype patients. Additionally, we also examined the relationship between PSPH expression and the clinicopathological characteristics of patients with gastric cancer such as invasive depth and distant metastasis (Table 2, *P* < 0.001 and *P* = 0.012, respectively). These results suggest that the differential expression of PSPH across such clinicopathological characteristics may be involved distinguished molecular targets as potential biomarker for prognosis prediction and a therapeutic target in gastric cancer.

**Table 2:** Correlation of clinical characteristcs of 234 gastric cancer patients with different expression of PSPH

Variables	PSPH expression		*P*
	Low (n = 108)	High (n = 126)	
Age (years)			
≤ 59	59 (54.6)	65 (51.6)	
> 59	49 (45.4)	61 (48.4)	0.642
Gender			
Male	67 (62.0)	85 (67.5)	
Female	41 (38.0)	41 (32.5)	0.386
Lauren classification			
Intestinal-type	23 (21.3)	41 (32.5)	
Diffuse-type	70 (64.8)	41 (32.5)	
Mixed	15 (13.9)	44 (35.0)	< 0.001
Invasion depth			
T1	21 (19.6)	4 (3.2)	
T2	7 (6.5)	16 (12.8)	
T3	41 (38.2)	52 (41.6)	
T4	38 (35.5)	53 (42.4)	< 0.001
Lymph node metastasis			
Negative	32 (30.2)	30 (24.6)	
Positive	74 (69.8)	92 (75.4)	0.343
Distant metastasis			
Negative	102 (94.4)	106 (84.1)	
Positive	6 (5.6)	20 (15.9)	0.012
Stage			
I + II	47 (43.5)	44 (35.2)	
III + IV	61 (56.5)	81 (64.8)	0.194

PSPH, phosphoserine phosphatase

### High PSPH level is a possible risk factor in gastric cancer

**Fig. 3:**
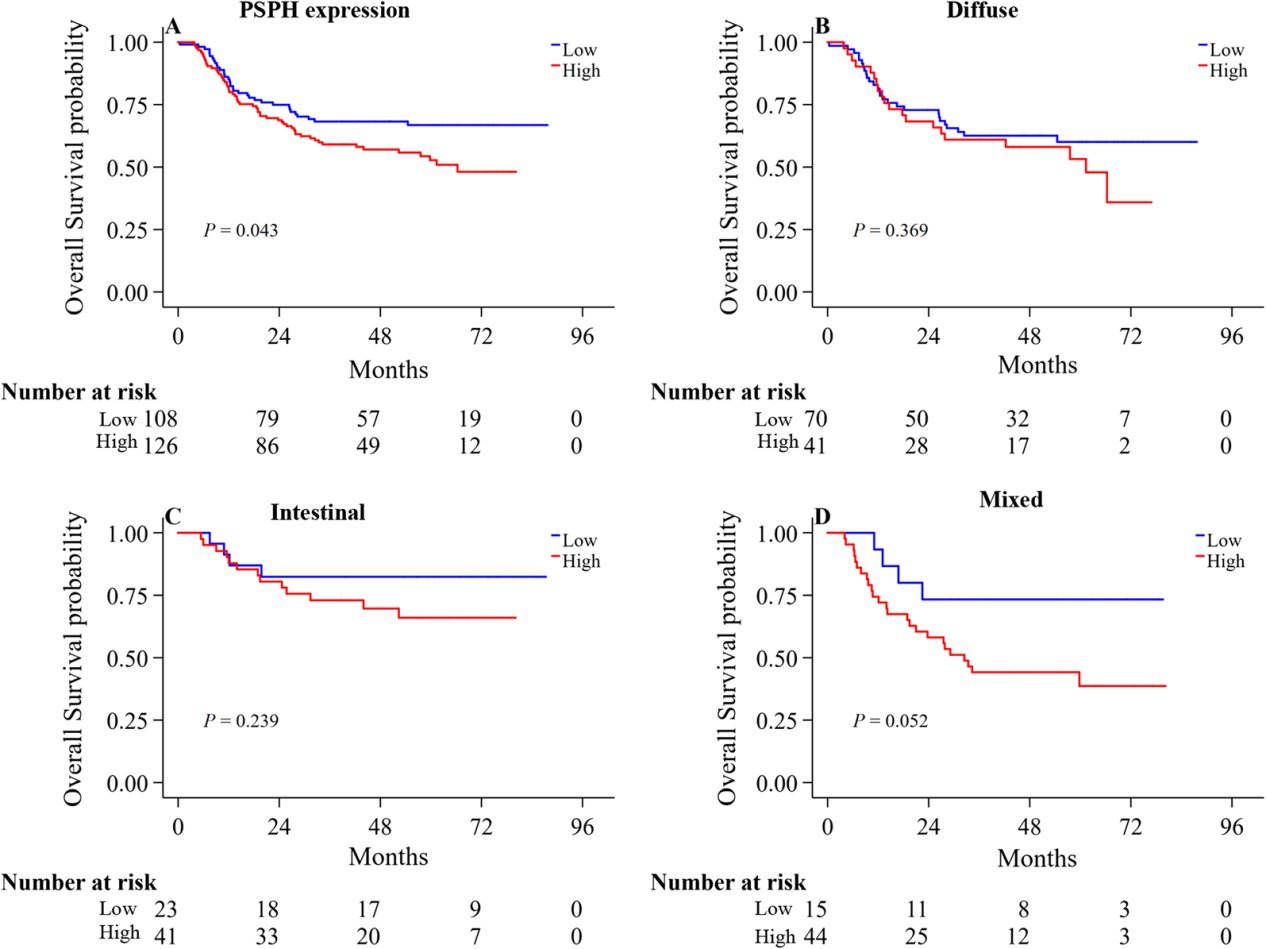
The Kaplan–Meier survival analysis of PSPH expression levels (**A**) further stratified by pathological sub-type: diffuse-type (**B**), intestinal-type (**C**) and mixed-type (**D**)

We conducted Kaplan–Meier survival analysis to elucidate the roles of PSPH expression in gastric cancer. The patients with high PSPH expression had a significantly inferior prognosis (Fig. 3A* P* = 0.043). Stratified with the pathological classification, we did not find any significance in overall survival according to the PSPH expression (Fig. 3B–D). Furthermore, a univariate Cox analysis identified PSPH expression as a risk factor associated with the prognosis in gastric cancer (Table 3). However, subsequent multivariate Cox analysis revealed that invasive depth and distant metastasis were independently associated with the prognosis in gastric cancer (Table 3).

**Table 3:** Univariate associations of clinicopathological characteristics and PSPH expression with OS in the 234 gastric patients

Variables	Univariate		Multivariate	
	HR (95% CI)	P	HR (95% CI)	P
Age				
≤ 59	1 (reference)			
> 59	1.23 (0.82–1.85)	0.313		
Gender				
Male	1 (reference)			
Female	1.01 (0.66–1.54)	0.986		
Lauren classification				
Intestinal-type	1 (reference)		1 (reference)	
Diffuse-type	1.81 (1.04–3.15)	0.037	1.32 (0.73–2.39)	0.346
Mixed	2.24 (1.23–4.08)	0.008	1.23 (0.66–2.29)	0.507
Invasion depth				
T1	1 (reference)		1 (reference)	
T2	2.33 (0.43–12.73)	0.328	4.08 (0.44–37.11)	0.212
T3	3.51 (0.83–14.9)	0.088	3.62 (0.45–28.67)	0.222
T4	14.94 (3.65–61.17)	< 0.001	10.55 (1.32–84.30)	0.026
Lymph node metastasis				
Negative	1 (reference)		1 (reference)	
Positive	3.79 (1.96–7.32)	< 0.001	1.08 (0.39–2.96)	0.868
Distant metastasis				
Negative	1 (reference)		1 (reference)	
Positive	5.74 (3.56–9.25)	< 0.001	2.61 (1.55–4.39)	< 0.001
Stage				
I + II	1 (reference)		1 (reference)	
III + IV	5.51 (3.06–9.93)	< 0.001	2.10 (0.76–5.77)	0.150
PSPH expression				
Low	1 (reference)		1 (reference)	
High	1.53 (1.01–2.34)	0.041	1.30 (0.81–2.08)	0.275

PSPH, phosphoserine phosphatase; OS, overall survival; HR, hazard ratio; CI, interval incidence

### PSPH is associated with immune cells in gastric cancer

**Fig. 4:**
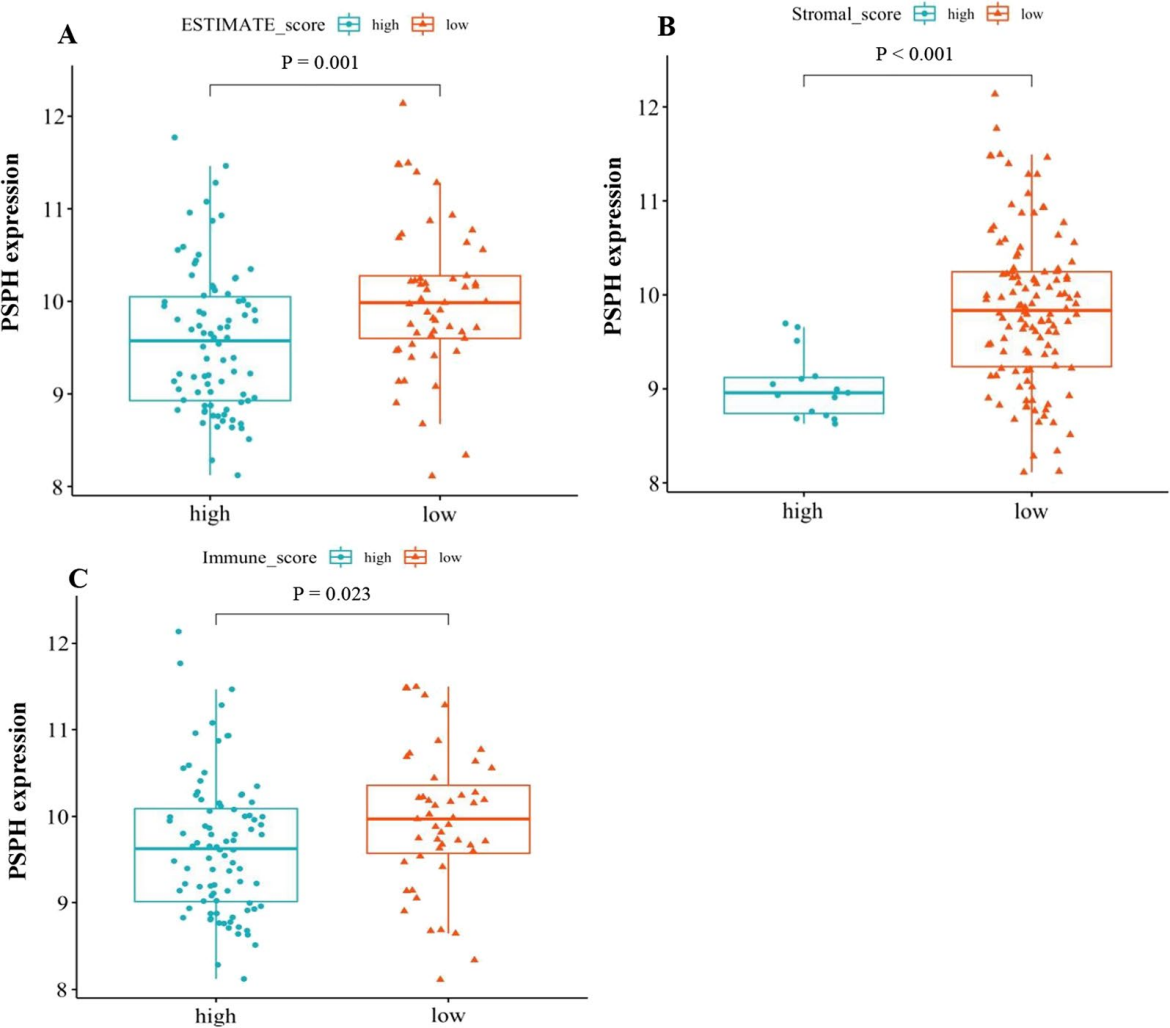
The relationship between PSPH expression levels and the density of immune score in patients with gastric cancer: the low density of ESTIMATE score (**A**), stromal score (**B**) and immune score (**C**) in the group with high PSPH expression

**Fig. 5:**
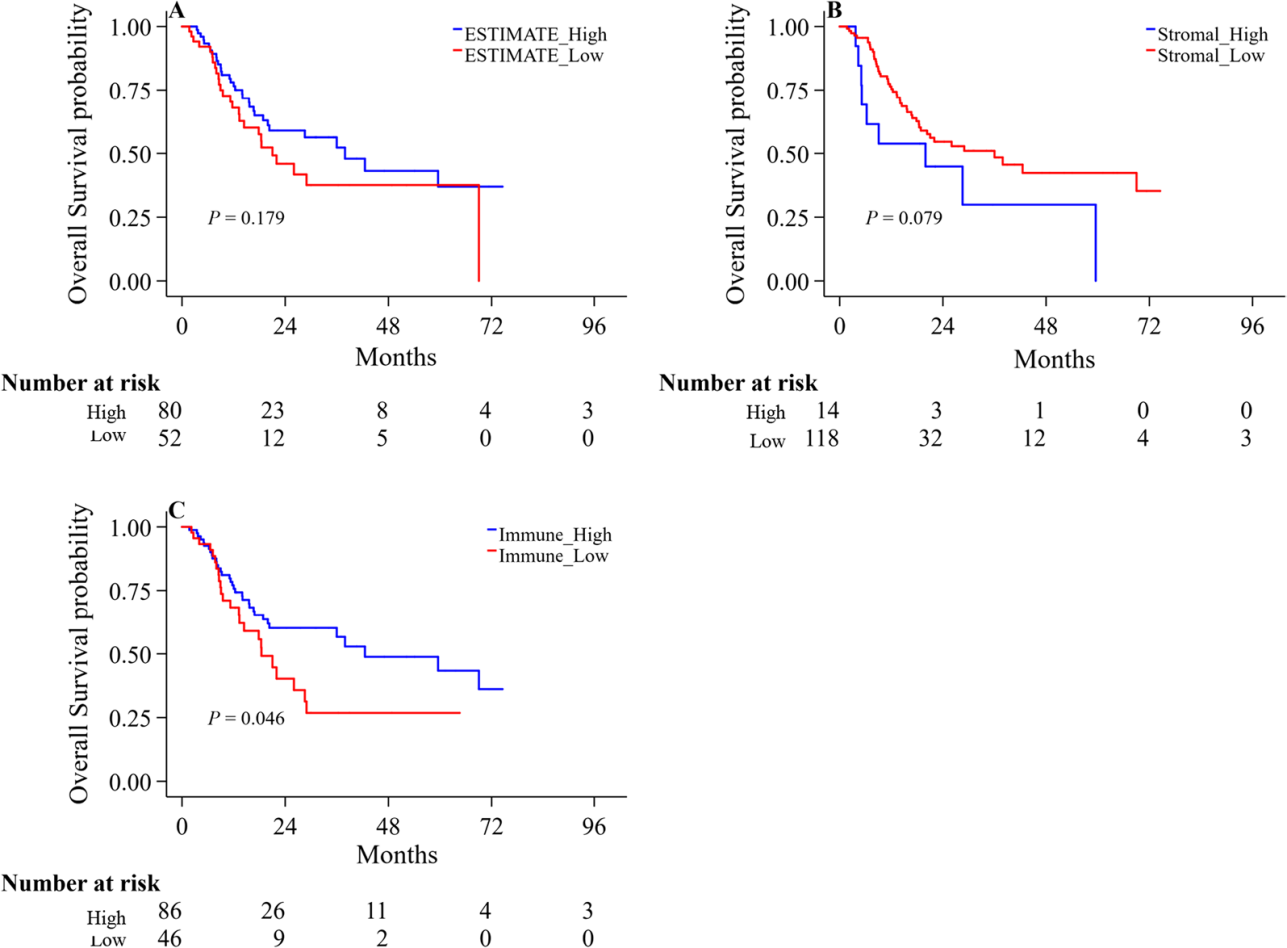
The Kaplan–Meier survival curves of patients with different density of immune cells: ESTIMATE score (**A**), stromal score (**B**) and immune score (**C**)

**Fig. 6:**
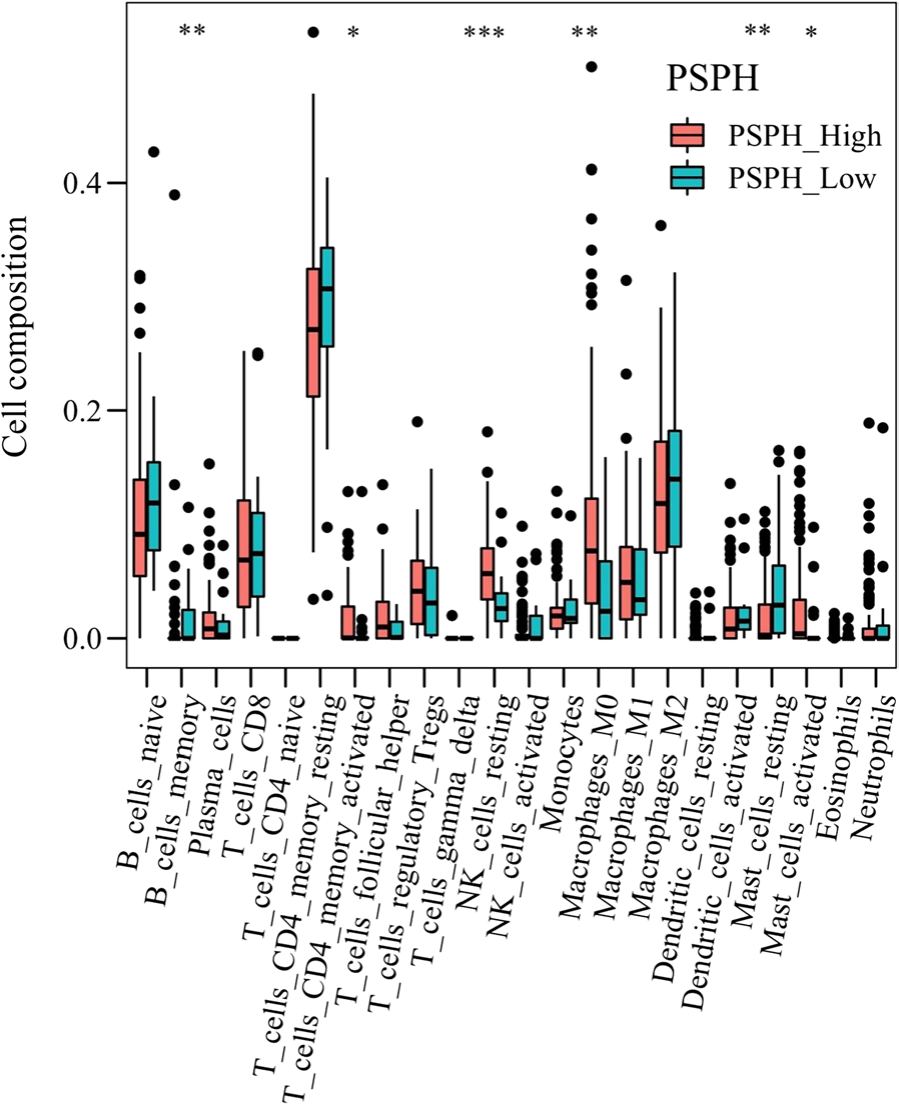
The distribution of 22 types of immune cells in the conditions of high and low expression of PSPH. *P < 0.05, **P = 0.01, ***P = 0.001 and ****P = 0.0001

**Fig. 7:**
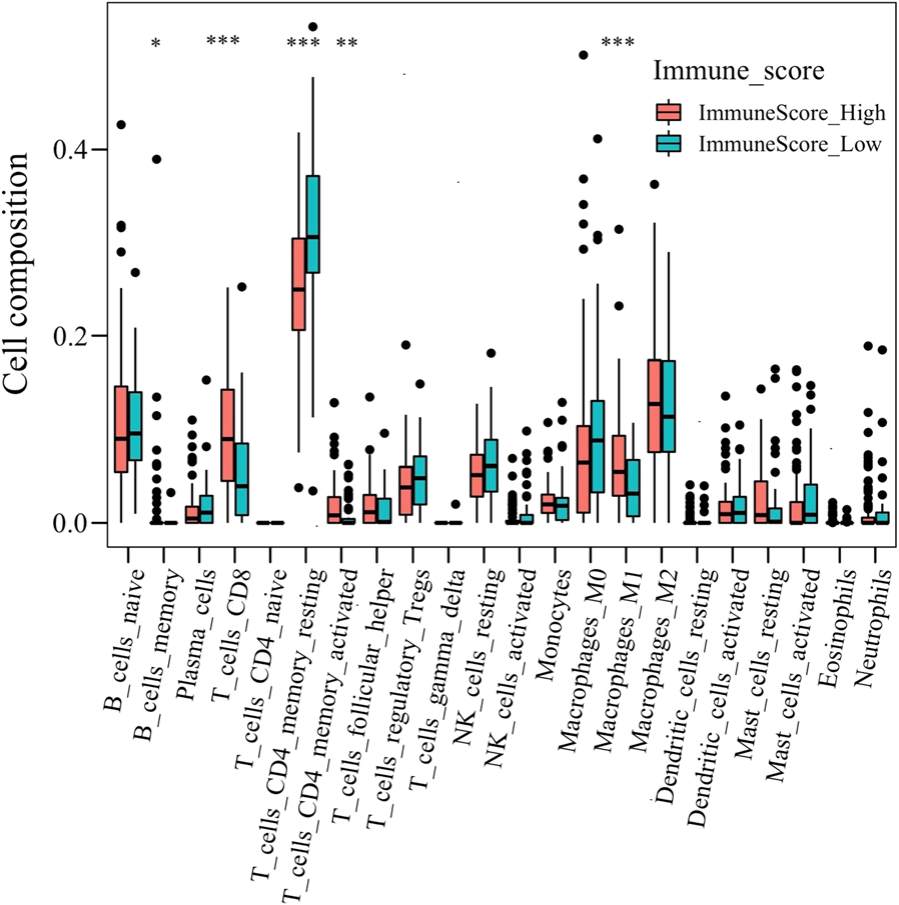
The distribution of 22 types of immune cells is shown in the high and low score of immune cells assessed by the ESTIMATE algorithm. *P < 0.05, **P = 0.01 and ***P = 0.001

To further understand the potential mechanism of action of PSPH in gastric cancer, we continued to perform the ESTIMATE analysis on this TCGA-STAD cohort data and calculated immune score in patients with gastric cancer, showing significant differences in the ESTIMATE, stromal and immune scores between the patients with high or low PSPH expression. All the scores were lower in patients with high expression of PSPH (Fig. 4A–C; *P* = 0.001, < 0.001 and 0.023, respectively). Moreover, low immune score was associated with poor prognosis (Fig. 5C *P* = 0.046), whereas no relationship was observed between ESTIMATE and stromal scores and prognosis (Fig. 5A, B). To comprehend more deeply immune cell subtype affecting prognosis, we analysed the proportion of 22 immune cells in the two groups by the Cibersort algorithm. It was observed that there were significant differences in memory B cells, activated CD4 memory T cells, resting NK cells, M0 macrophages and resting and activated mast cells between the two groups with high or low PSPH expression (Fig. 6, *P* < 0.05) and in memory B cells, CD8 T cells, resting memory CD4 T cells, activated memory CD4 T cells and M1 macrophages between the two groups with high or low score of immune cells (Fig. 7, *P* < 0.05). These findings showed that PSPH expression was associated with the immune cells in patients with gastric cancer, potentially explaining the poor survival.

## Discussion

It was observed in the study that PSPH expression was higher in patients with gastric cancer than in normal individuals. Patients with high expression of PSPH had poor survival. More importantly, high expression of PSPH was found to be significantly associated with the low score of immune cells, mainly activated memory CD4 T cells, which conferred poor survival. These results indicated that differential expression of PSPH may contribute to survival of patients with gastric cancer. Furthermore, we speculated that the underlying mechanism of action was due to the dysregulation of PSPH–immune axis, which might provide a new treatment regimen for patients with gastric cancer.

PSPH is associated with the development of various cancers and is a promising prognostic biomarker in different cancers such as advanced colorectal cancer [17], thyroid cancer [18] and melanoma [19]. Similarly, we found high PSPH expression in most of the patients with gastric cancer who had a dismal survival. These findings indicated that differential expression of PSPH could play an important role in the carcinogenesis of gastric cancer. Recently, Kim et al. have reported that specific subtypes of gastric cancer are characterised by differential sensitivity to immunotherapy such as PD-L1 blockade [20], and may also display different levels of PSPH expression across pathological subtypes. Autophagy phenomena is highly associated with PSPH expression in cancers [21], implicating that PSPH expression is important in gastric cell physiology and the interaction with Helicobacter, as evident from genetic polymorphisms in the population that predispose to the disease [22]. Apart from these mechanisms, it have been reported in many previous studies that the action PSPH in cancers are 5-fluorouracil-induced increased accumulation of reactive oxygen species through PSPH-mediated serine synthesis in colorectal cancer [23], inhibition of apoptosis in hepatocellular cancer, promotion of osteoclastogenesis in bone metastatic breast cancer [24] and notably, EGFR amplification in lung adenocarcinoma which was associated with PSPH overexpression [25].

Recent studies reported that alteration of tumour immune microenvironment in gastric cancer is a pivotal strategy for predicting the immunotherapeutic responses and prognosis, which might provide new treatment strategies for patients [26, 27]. To explore the possible mechanism of PSPH affecting the survival, we analysed the relationship between PSPH expression and ESTIMATE, stromal and immune scores and found that the patients with higher PSPH expression conversely exhibited a lower ESTIMATE, stromal and immune scores. In particular, patients with lower immune score had a poor prognosis. Furthermore, through the Cibersort algorithm, we found that the amount of resting NK cells, activated memory CD4 T cells and M0 macrophages was high in high PSPH expression group, whereas that of memory B cells and activated or resting mast cells was low. It confirmed that PSPH may participate in the regulation of the tumour immune microenvironment in gastric cancer.

The tumour immune microenvironment contains multiple complex components capable of suppressing and promoting tumour metastasis and growth [28]. Substantial evidence has already indicated that cellular metabolism is involved in immune cell functions. PSPH-mediated serine synthesis is a well-known process associated with various cellular responses. It is upregulated in cancer cells as a mechanism contributing to enhanced nucleotide and amino acid synthesis with regard to cell proliferation and antioxidant production, and it is a potential novel therapeutic target for treating cancers [29–31]. T cells and macrophages are the most abundant cells among all types of immune cells with varying functions. Noga et al. reported that serine biosynthesis is necessary for T cell expansion in acquired immune responses [32]. On the other hand, in certain animal models, excessive serine management can suppress inflammatory responses by increasing glutathione synthesis [33]. Recent experimental studies have shown that macrophages promote the cancer progression by releasing various cytokines, including chemokines and inflammatory factors [34], namely grouped into M1 and M2. However, these two sub-types have opposite effects on tumour progression. M2 macrophages are mainly conducive for angiogenesis and stimulate tumour cell metastasis [35]; by the contrast, M1 macrophages are the tumour suppressors as per the effects of pro-inflammatory and cytotoxic cell expression. Along with the upregulation of PSPH, the contents of M0 macrophages, resting NK cells and activated memory CD4 T cells also increased. Wu et al. have recently reported that patients with gastric cancer with high expression of ADAMTS12 had an unfavourable prognosis, and interestingly, the content of M0 macrophages was also increased [36]. In another study, it was demonstrated that high-risk in patients with lung cancer was associated with significantly high levels of activated memory CD4 T cells, resting NK cells and M0 macrophages [37]. Through data analysis, we found that patients with gastric cancer with high expression of PSPH and low immune score had poor prognosis because of a high amount of M0 macrophages and a low amount of M1 macrophages, which was consistent with the previous study [38]. Collectively, this study demonstrated that the metabolic gene PSPH could affect the density of immune cells in patients with gastric cancer, and it could be an important clue for deciding treatment regimens.

Moreover, this study revealed that the mechanism of action of PSPH as an indicator of poor outcome in gastric cancer was associated with immune cell infiltrations. This indicated that PSPH could affect the infiltration of immune cells in gastric cancer. Further studies are warranted to explore and confirm the mechanism of PSPH in inhibiting immune cells in vitro and in vivo.

## Supplementary Information

**Additional file 1.** The variation in PSPH mRNA expression is exhibited within and across 32 cancer types using TCGA RNA-Seq data.

**Additional file 2.** Summary of TCGA samples surveyed in this study.

## Data Availability

The key raw data used and/or analyzed during the present study are available within the manuscript and its supplementary files and have also been deposited into the Research Data Deposit (https://www.researchdata.org.cn/), with the approval number of RDDA2021002100.
