# Supplementary material for: Phosphoserine phosphatase as a prognostic biomarker in patients with gastric cancer and its potential association with immune cells
Source: BMC Gastroenterol. 2022 Jan 3;22:1. doi: 10.1186/s12876-021-02073-0 (PMC8722028; doi:10.1186/s12876-021-02073-0)
Supplement: Supplementary file 1 — Additional file 1. The variation in PSPH mRNA expression is exhibited within and across 32 cancer types using TCGA RNA-Seq data. [file 12876_2021_2073_MOESM1_ESM.docx]

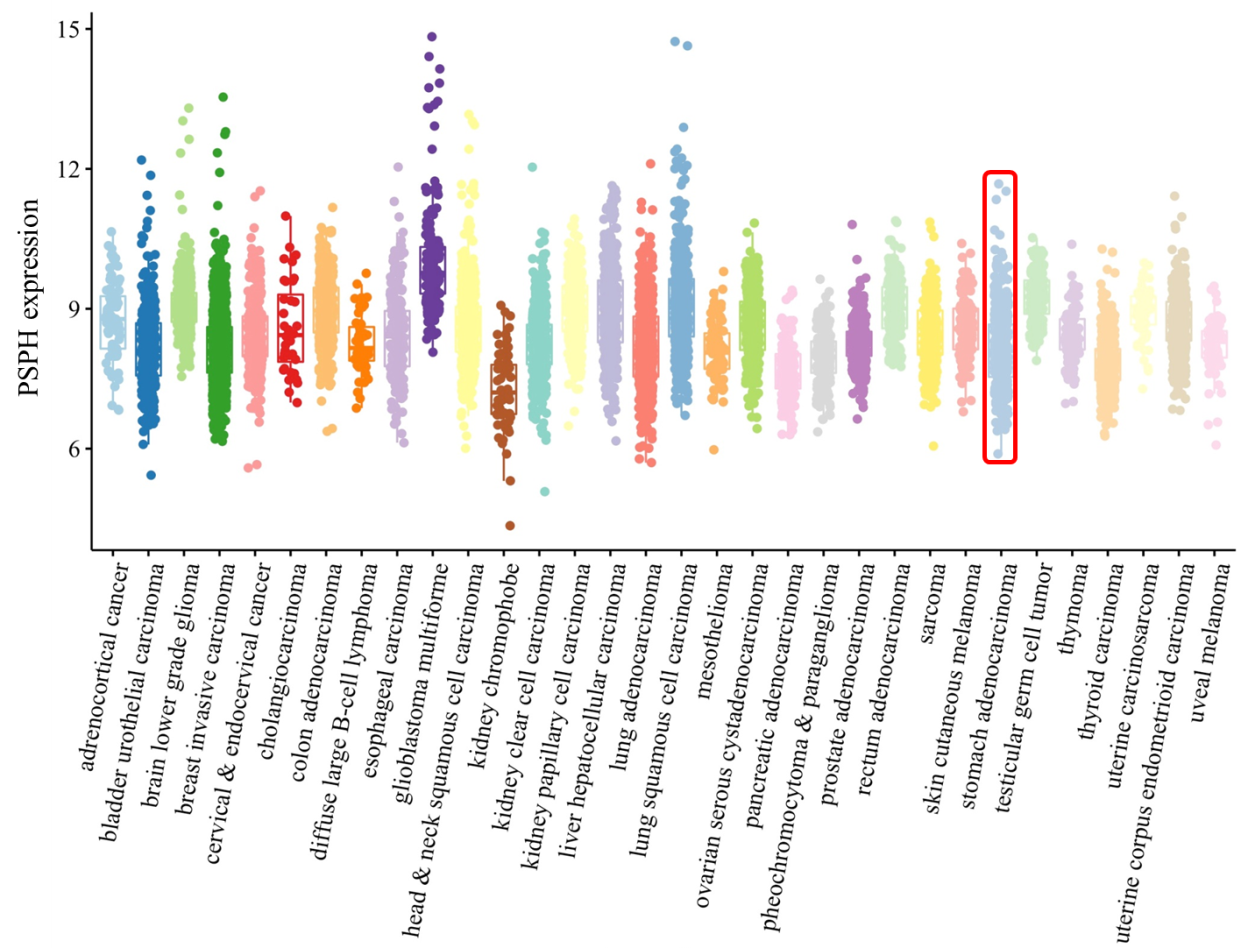


**Supplementary Figure S1**. The variation in PSPH mRNA expression is exhibited within and across 32 cancer types using TCGA RNA-Seq data. The middle line in the box is the median, the bottom and top of the box are the first and third quartiles, and the whiskers extend to 1.5 IQR of the lower quartile and the upper quartile, respectively. The red box indicates gastric cancer.

**Abbreviation**: PSPH, phosphoserine phosphatase; IQR, interquartile range.
